# Identification of hub genes and key signaling pathways by weighted gene co-expression network analysis for human aortic stenosis and insufficiency

**DOI:** 10.3389/fcvm.2023.857578

**Published:** 2023-08-09

**Authors:** Yang Yang, Bing Xiao, Xin Feng, Yue Chen, Qunhui Wang, Jing Fang, Ping Zhou, Xiang Wei, Lin Cheng

**Affiliations:** ^1^ Division of Cardiothoracic and Vascular Surgery, Tongji Hospital, Tongji Medical College, Huazhong University of Science and Technology, Wuhan, China; ^2^ Institute of Organ Transplantation, Tongji Hospital, Tongji Medical College, Huazhong University of Science and Technology, Wuhan, China; ^3^ Key Laboratory of Organ Transplantation, Ministry of Education, Wuhan, China; ^4^ NHC Key Laboratory of Organ Transplantation, Chinese Academy of Medical Sciences, Wuhan, China; ^5^ Key Laboratory of Organ Transplantation, Chinese Academy of Medical Sciences, Wuhan, China; ^6^ Department of Obstetrics and Gynecology, Peking University People's Hospital, Peking University, Beijing, China

**Keywords:** aortic valve stenosis, aortic valve insufficiency, heart failure, WGCNA, co-expression modules, hub genes

## Abstract

**Background:**

Human aortic valve stenosis (AS) and insufficiency (AI) are common diseases in aging population. Identifying the molecular regulatory networks of AS and AI is expected to offer novel perspectives for AS and AI treatment.

**Methods:**

Highly correlated modules with the progression of AS and AI were identified by weighted genes co-expression network analysis (WGCNA). Gene Ontology (GO) and Kyoto Encyclopedia of Genes and Genomes (KEGG) enrichment analyses were performed by the clusterProfiler program package. Differentially expressed genes (DEGs) were identified by the DESeqDataSetFromMatrix function of the DESeq2 program package. The protein-protein interaction (PPI) network analyses were implemented using the STRING online tool and visualized with Cytoscape software. The DEGs in AS and AI groups were overlapped with the top 30 genes with highest connectivity to screen out ten hub genes. The ten hub genes were verified by analyzing the data in high throughput RNA-sequencing dataset and real-time PCR assay using AS and AI aortic valve samples.

**Results:**

By WGCNA algorithm, 302 highly correlated genes with the degree of AS, degree of AI, and heart failure were identified from highly correlated modules. GO analyses showed that highly correlated genes had close relationship with collagen fibril organization, extracellular matrix organization and extracellular structure organization. KEGG analyses also manifested that protein digestion and absorption, and glutathione metabolism were probably involved in AS and AI pathological courses. Moreover, DEGs were picked out for 302 highly correlated genes in AS and AI groups relative to the normal control group. The PPI network analyses indicated the connectivity among these highly correlated genes. Finally, ten hub genes (*CD74*, *COL1A1*, *TXNRD1*, *CCND1*, *COL5A1*, *SERPINH1*, *BCL6*, *ITGA10*, *FOS*, and *JUNB*) in AS and AI were found out and verified.

**Conclusion:**

Our study may provide the underlying molecular targets for the mechanism research, diagnosis, and treatment of AS and AI in the future.

## Introduction

Heart failure is a severe terminal stage of all kinds of cardiovascular diseases, which include hypertension (1), coronary heart disease (2), myocardial infarction (3), valvular heart disease (4), and cardiomyopathy (5). As the important cause of heart failure, valvular heart diseases consist of stenosis or insufficiency with specific pathophysiology among the four cardiac valves (aortic valves, mitral valves, tricuspid valves and pulmonary valves) (6). In the globe, valvular heart diseases have increasingly become the important contributor to cardiovascular morbidity and mortality according to the epidemiologic studies, which have resulted in serious social burden and economical cost on valvular heart diseases diagnosis and treatment (7). The prevalence of valvular heart diseases gradually increases with age of clinical patients (8). In terms of aortic valve lesion, aortic valve stenosis (AS) and aortic valve regurgitation (AR) are the highly popular valve lesions among various valvular heart diseases (9), the morbidities of AS and AR were 0.7% and 0.2% with the age 55–64 years, 1% and 1.3% for the age-bracket of 65–74 years, and 2% and 2.8% after 75 years old, respectively (8). Thus, due to the large aging population around the world, the aortic valvular heart diseases are still the important public health problem.

AS and AR are a kind of common aortic valve diseases, characterized by aortic valve opening area reduction or aortic valve insufficiency (AI), respectively. Currently, numerous studies have reported the pathological features and molecular mechanisms about aortic valve damage (10–13). The well-known etiologies for AS include aortic valve degeneration, rheumatic aortic stenosis, congenital valve defects, systemic inflammatory diseases, endocarditis, and many other conditions (10). Whereas, the major causes of AI are made up of various pathological changes of aortic valves, such as leaflet abnormalities, rheumatic fever, myxomatous degeneration, infective endocarditis, etc (12). Although, the current available reports have uncovered the molecular mechanisms for pathological processes of AS and AI, which include but not limit to fibro-calcific remodeling, osteogenic differentiation, lipid accumulation, inflammation, angiogenesis and hemorrhage, disorganization and remodeling of the valvular extracellular matrix (ECM) (10, 13). The vital molecules for the regulation and indication of AS and AI pathological courses still need to be further investigated. Hence, using high throughput sequencing techniques and identifying the key regulatory or indicative molecules for AS and AI may provide a feasible strategy for the diagnosis and treatment of AS and AI from the microscopic molecular viewpoints.

In this study, we analysed the expression profiles of human aortic valve samples of aortic valve stenosis (AS) and aortic insufficiency (AI) by systematic bioinformatics approaches of weighted gene co-expression network analysis (WGCNA). We constructed the gene co-expression modules by WGCNA algorithm and screened the highly correlated modules with the degree of AS, degree of AI, and heart failure, which included orange, steelblue, darkgreen, and grey60 modules. Furthermore, we selected highly correlated genes in indicated modules and performed Gene Ontology (GO) and Kyoto Encyclopedia of Genes and Genomes (KEGG) pathway enrichment analyses. The differentially expressed genes (DEGs) were identified and intersected with those 30 genes possessing high connectivity among the highly correlated genes from various modules to screen out hub genes. Finally, the mRNA expression values of ten hub genes (*CD74*, *COL1A1*, *TXNRD1*, *CCND1*, *COL5A1*, *SERPINH1*, *BCL6*, *ITGA10*, *FOS*, and *JUNB*) were validated by analyzing results of high throughput RNA sequencing from AS and AI aortic valve samples and by examining the mRNA expression levels of human AS and AI aortic valve tissues. These results may provide an avenue for the diagnosis and treatment of AS and AI in the future.

## Materials and methods

### High-throughput data acquisition and preprocessing

The high-throughput RNA-sequencing datasets were acquired from the public Gene Expression Omnibus (GEO) database with the accession number GSE153555, which contained the gene expression data from 5 human normal control (NC) aortic valves, 5 human aortic stenosis (AS) aortic valves, and 5 human aortic insufficiency (AI) aortic valves, each individual contained 2 biologically repeated aortic valve high-throughput RNA-sequencing results. The gene expression Fragments Per Kilobase of transcript per Million mapped reads (FPKM) values and count values were analysed by R software (Version 4.1.2). Clinical traits, including age, sex, body mass index (BMI), degree of AS and AI, left ventricular ejection fraction (LVEF), and disease history (diabetes, hypertension, coronary heart disease, and heart failure), for each sample were collected from the Series Matrix Files in the GEO database with GSE153555 number (14). The average gene expression FPKM values of 30 samples were calculated and ranked by size, and the top 6,000 genes with the highest average expression were screened out and used for weighted gene co-expression network analysis (WGCNA) computation. The FPKM values of the 6,000 genes from 30 samples were subjected to log_2_(FPKM + 1) conversion followed by samples hierarchical clustering to eliminate 2 outlier samples (GSM4647040 and GSM4647041) using the hclust function in the R software (Version 4.1.2).

### Co-expression module construction of AS and AI by WGCNA algorithm

The WGCNA co-expression module construction was conducted as previously described (15). The soft threshold β power value for WGCNA module construction was computed by pickSoftThreshold function in the WGCNA program package. The adequate β value 16 was picked out once Scale Free Topology Model Fit, signed *R*^2^ value was ≥0.8. Then, the adjacency matrix and topological overlap matrix (TOM) were constructed using the power value 16. The co-expression modules were constructed and merged for those modules with similar expression profiles by step-by-step network construction methods. The correlation analysis among the indicated modules were performed by calculating the eigengenes, which were defined as the principal component 1 (PC1) of principal component analysis (PCA) for gene expression values in the indicated modules.

### Correlation analysis between co-expression modules and clinical traits

The correlation analyses between modules and clinical traits were performed using the eigengenes of the corresponding modules and clinical traits data to screen out the highly correlated modules with indicated clinical traits. To screen the highly correlated genes in AS and AI pathological courses, these modules relevant to degree of AS, degree of AI, and heart failure were further analysed. These modules with correlation coefficient more than 0.6 and *P* value less than 0.05 were regarded as highly correlated modules. The correlation coefficients of 6,000 genes expression values and indicated clinical traits were defined as gene significance (GS). For the associations of each module with the genes involved in the WGCNA process, module membership (MM) was defined as the correlation of module eigengenes and gene expression levels. The scatterplot of Gene Significance *vs.* Module Membership in the indicated module was plotted. These scatterplots with correlation coefficients more than 0.5 and *P* value less than 0.05 were selected. These 302 genes in indicated scatterplot with MM > 0.8 and GS > 0.8 were regarded as the highly correlated genes with corresponding module or trait, respectively, and were selected for subsequent analysis.

### Gene ontology (Go) and Kyoto encyclopedia of genes and genomes (KEGG) enrichment analyses

We performed GO and KEGG enrichment analyses for the highly correlated genes identified by the above procedures using the clusterProfiler program package. Firstly, the ENSEMBL number for each gene was transformed into the ENTREZID number using the bitr function. GO analyses including biological processes (BP), molecular functions (MF), and cellular components (CC) terms were conducted. These terms in GO and KEGG enrichment analyses with *P* value less than 0.05 were screened out and considered as significant terms in AS and AI pathological processes. The top ten terms in GO and KEGG enrichment analyses were selected for visualization and further analyses.

### Identification of differentially expressed genes (DEGs)

The DEGs analysis using the high throughput RNA-sequencing data was performed to evaluate the gene expression situation among the indicated groups using the DESeqDataSetFromMatrix function of the DESeq2 program package. Firstly, the results of DEGs analyses for total genes in high throughput RNA-sequencing data were obtained from the AS and AI group. The indicated DEGs results for the 302 highly correlated genes were screened out. These genes in AS or AI group with |log_2_FoldChange| ≥ 1 and adjust *P* value <0.05 relative to the normal control group, were considered as the DEGs. These genes with log_2_FoldChange ≥ 1 were defined as upregulated (UP) genes. These genes with log_2_FoldChange ≤ −1 were defined as downregulated (DOWN) genes. These genes with −1 < log_2_FoldChange < 1 were defined as unchanged (NOT) genes. The volcano plots and heat maps were plotted to visualize DEGs.

### Protein-protein interaction (PPI) network analysis

PPI network analysis was performed using an online network tool STRING (https://cn.string-db.org/, version 11.5) (16). The 302 highly correlated genes were imported into STRING. The TSV file contained 302 highly correlated genes was downloaded and the PPI network was visualized by Cytoscape software (version 3.9.0) (17). The CytoHubba plug-in was used for identifying genes with high connectivity ranked by Betweenness (18). The top 30 genes with highest connectivity were picked out for further analysis. The DEGs of AS and AI group were intersected concurrently with the top 30 genes from PPI network analysis to identify these genes with significantly changed expression and high connectivity. These genes (*CD74*, *COL1A1*, *TXNRD1*, *CCND1*, *COL5A1*, *SERPINH1*, *BCL6*, *ITGA10*, *FOS*, and *JUNB*) that met the above conditions were identified as the hub genes.

### Human aortic valves sample collection and grouping

Human aortic valve samples were obtained from patients with pure AS or AI. These aortic valves samples from heart transplantation receptors or aortic dissection patients without definite lesions in aortic valves were used as normal control (NC) and mild aortic valve samples. A total of 35 aortic valve samples including 5 samples from normal control (NC) patient, 15 samples from aortic stenosis (AS) patient and 15 samples from aortic insufficiency (AI) patient were included in this study. Doppler echocardiography was used for evaluation of AR or AS severity (19, 20). The specimens were classified into 4 groups containing normal control, mild, moderate, and severe. The characteristics of aortic valves used in this study are shown in Table 1. All procedures involving human aortic valves samples conformed to the principles outlined in the Declaration of Helsinki. Exemption from informed consent for patients and human sampling procedures were approved by the Human Research Ethics Committees of Tongji Hospital of Huazhong University of Science and Technology (21).

**Table 1:** Characteristics of clinical samples used in this study.

Group	Age (years)	Sex	Body weight (kg)	Height (cm)	Degree of aortic Valve lesion	LA diameter (mm)	LV diameter (mm)	LVEF
NC-1	67	M	68	170	Normal	32	46	70%
NC-2	30	F	50	168	Normal	26	37	71%
NC-3	43	F	60	163	Normal	28	40	68%
NC-4	51	M	69	173	Normal	30	46	72%
NC-5	44	M	70	170	Normal	28	43	69%
AS-1	51	M	70	173	Mild AS	29	48	68%
AS-2	34	F	55	157	Mild AS	30	48	72%
AS-3	57	F	56	158	Mild AS	33	44	69%
AS-4	45	F	60	163	Mild AS	29	43	71%
AS-5	81	M	68	167	Mild AS	31	46	74%
AS-6	55	M	71	168	Moderate AS	28	45	71%
AS-7	54	F	65	163	Moderate AS	30	46	68%
AS-8	68	F	75	170	Moderate AS	35	48	68%
AS-9	58	F	56	157	Moderate AS	34	44	68%
AS-10	56	M	70	170	Moderate AS	31	53	60%
AS-11	48	F	46	156	Severe AS	25	38	63%
AS-12	59	F	76	163	Severe AS	35	48	68%
AS-13	41	M	75	171	Severe AS	36	58	67%
AS-14	70	M	80	168	Severe AS	36	47	67%
AS-15	50	F	46	151	Severe AS	27	43	60%
AI-1	56	M	70	173	Mild AI	25	49	67%
AI-2	60	F	56	156	Mild AI	30	52	65%
AI-3	53	F	46	153	Mild AI	26	48	66%
AI-4	66	M	68	168	Mild AI	29	47	68%
AI-5	50	F	55	160	Mild AI	26	38	70%
AI-6	58	F	60	160	Moderate AI	35	52	65%
AI-7	51	F	56	156	Moderate AI	36	51	68%
AI-8	59	F	46	153	Moderate AI	27	43	74%
AI-9	60	F	66	159	Moderate AI	36	58	63%
AI-10	60	M	68	170	Moderate AI	33	70	42%
AI-11	69	M	67	167	Severe AI	37	70	45%
AI-12	59	M	73	172	Severe AI	49	60	63%
AI-13	61	M	70	169	Severe AI	33	60	61%
AI-14	41	M	95	181	Severe AI	30	64	55%
AI-15	58	F	80	170	Severe AI	35	78	50%

A total of 35 aortic valve samples were included in the study, with 5 from normal control (NC) patients, 15 from aortic stenosis (AS) patients, and 15 from aortic insufficiency (AI) patients. F, female; M, male; LA, left atrial; LV, left ventricular; LVEF, left ventricular ejection fraction.

### Real-time PCR

Real-time PCR analyses of mRNA levels in human aortic valve samples were performed as previously described (21). Briefly, the total RNA was extracted from 5 normal control aortic valve samples, 15 AS aortic valve samples, and 15 AI aortic valve samples using TRIzol reagent (15596018, Thermo Fisher Scientific) and reverse transcribed into cDNA using the Transcriptor HiScript III RT SuperMix for qPCR (+gDNA wiper) (R323-01, Vazyme). The quantitative analyses of ten hub genes mRNA expression levels were determined by real-time PCR assay using SYBR (Q311-02, Vazyme). Glyceraldehyde-3-phosphate dehydrogenase (*GAPDH*) was used for the internal reference. Primers used in this study were listed in Table 2.

**Table 2:** Primers for the real-time PCR assays in this study.

Gene	Species		Sequence 5′-3′
*CD74*	Human	F	CAGCGCGACCTTATCTCCAA
		R	GGTACAGGAAGTAGGCGGTG
*COL1A1*	Human	F	AAGAACAGCGTGGCCTACAT
		R	TTCAATCACTGTCTTGCCCCA
*TXNRD1*	Human	F	TGGCCATTGGAATGGACGAT
		R	TGGACCCAGTACGTGAAAGC
*CCND1*	Human	F	GAGTGATCAAGTGTGACCCG
		R	CAGATGTCCACGTCCCGC
*COL5A1*	Human	F	ACAACAACCCCTACATCCGC
		R	TGACGCTTCACCGAAGTCAT
*SERPINH1*	Human	F	CCTCTCGAGCGCCTTGAAAA
		R	CTGACATGCGTGACAAGTCG
*BCL6*	Human	F	TTTCCGGCACCTTCAGACTC
		R	TGCACCTTGGTGTTGGTGAT
*ITGA10*	Human	F	AGACCCGGCCTATCCTCATC
		R	TTTCTTATGGGCAAAGAAGCCA
*FOS*	Human	F	GGAGGGAGCTGACTGATACAC
		R	ATCAGGGATCTTGCAGGCAG
*JUNB*	Human	F	GTCAAAGCCCTGGACGATCT
		R	TTGGTGTAAACGGGAGGTGG
*GAPDH*	Human	F	CATCACCATCTTCCAGGAGCGAGA
		R	TGCAGGAGGCATTGCTGATGATCT

### Statistical analysis

Statistical analysis was conducted by SPSS 23.0 software. All data are presented as the mean ± SD. Non-parametric Kruskal-Wallis H test was used for comparisons among multiple groups. *P* value <0.05 was considered to be statistically significant.

## Results

### Construction of WGCNA co-expression modules

**Figure 1:**
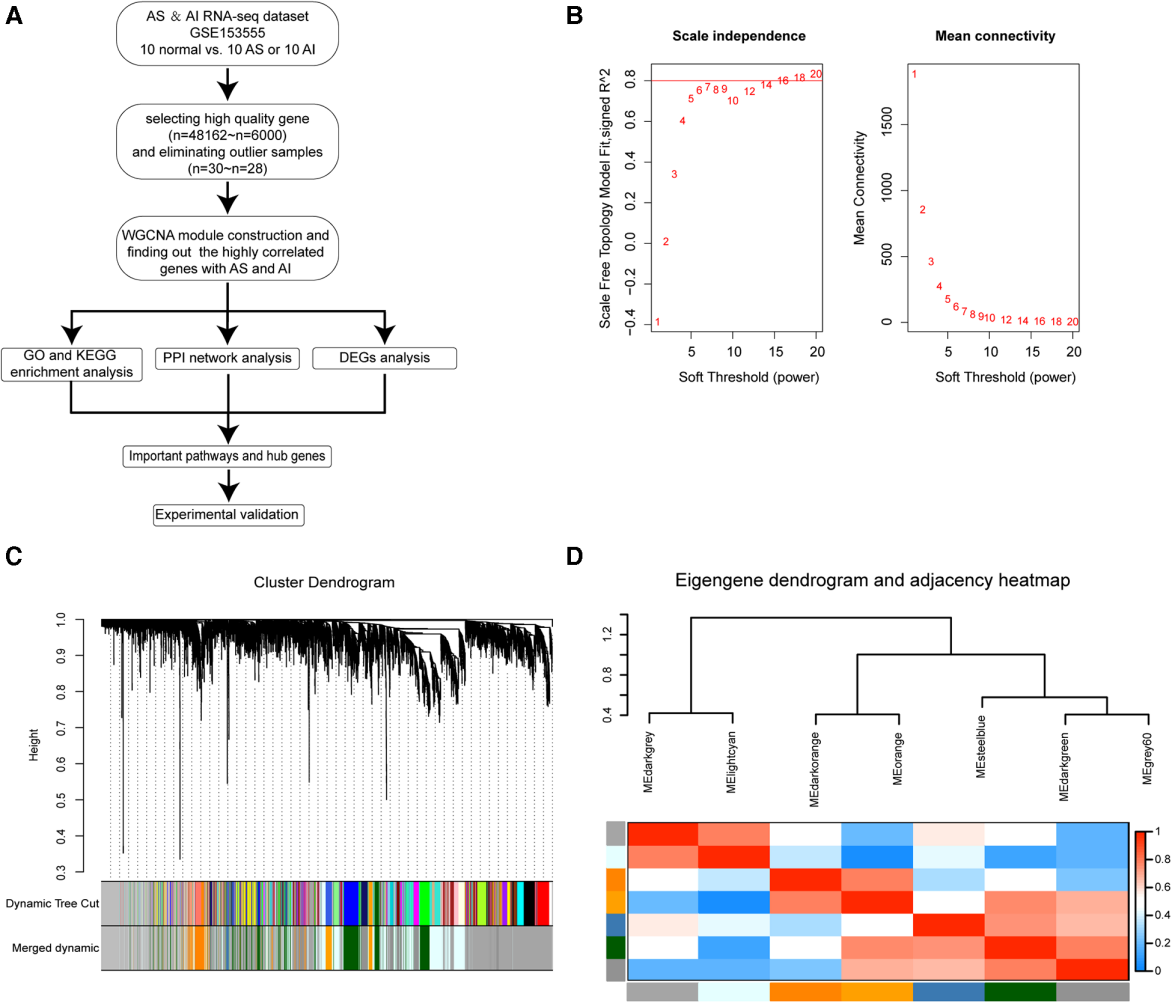
Construction of WGCNA co-expression modules. (**A**) The flow diagram for our WGCNA modules construction procedure in this study. (**B**) Analysis of scale-independence index (left panel) and mean connectivity for various soft threshold β power values, the most suitable β power value 16 was screened out. (**C**) Clustering dendrograms of 6,000 genes and module construction and merging according to the similar expression profiles by WGCNA algorithm. Total of eight co-expression modules were obtained including darkgrey, lightcyan, darkorange, orange, steelblue, darkgreen, grey60, and grey modules. The grey module is reserved for unassigned genes. (**D**) Cluster analysis for eigengenes dendrogram (Top) and correlation degree heatmap (Bottom) of eigengenes in each co-expression modules.

The data analysis process used in this study was depicted in the flow diagram (Figure 1A). High throughput RNA-sequencing data were preprocessed using the R software (Version 4.1.2). The average FPKM expression values for a total of 48,162 genes were calculated and ranked by size. The top 6,000 genes with the highest average FPKM expression values in the datasets of the 30 samples were chosen for WGCNA computation. Sample hierarchical clustering was performed with hclust function and the height 60 was set as the threshold to screen outlier samples (Figure S1A). Two outlier samples GSM4647040 and GSM4647041 were identified and eliminated from all samples (Figure S1A). Before network construction and module detection, the clinical traits related to the sample dendrograms were visualized as the heatmap in Figure S1B. Finally, 28 samples with 6,000 genes were selected for WGCNA module construction. To screen out the suitable soft threshold power value used for WGCNA algorithm, we set an indicated range for power values and the power value 16 was picked out by pickSoftThreshold function in WGCNA package (Figure 1B). The WGCNA module construction was conducted, and those modules with similar expression profiles were merged. As depicted by the gene dendrograms, total eight modules were finally constructed, which included darkgrey (1,978 genes), lightcyan (1,158 genes), darkorange (242 genes), orange (170 genes), steelblue (35 genes), darkgreen (706 genes), grey60 (649 genes), and grey (1,062 genes) modules (Figure 1C). Those genes uncorrelated with other modules were assigned to grey module. The eigengene dendrogram and eigengene adjacency heatmap were plotted to exhibit the associations among the modules by eigengenes (Figure 1D).

### Identification of highly correlated modules connected with AS or AI

**Figure 2:**
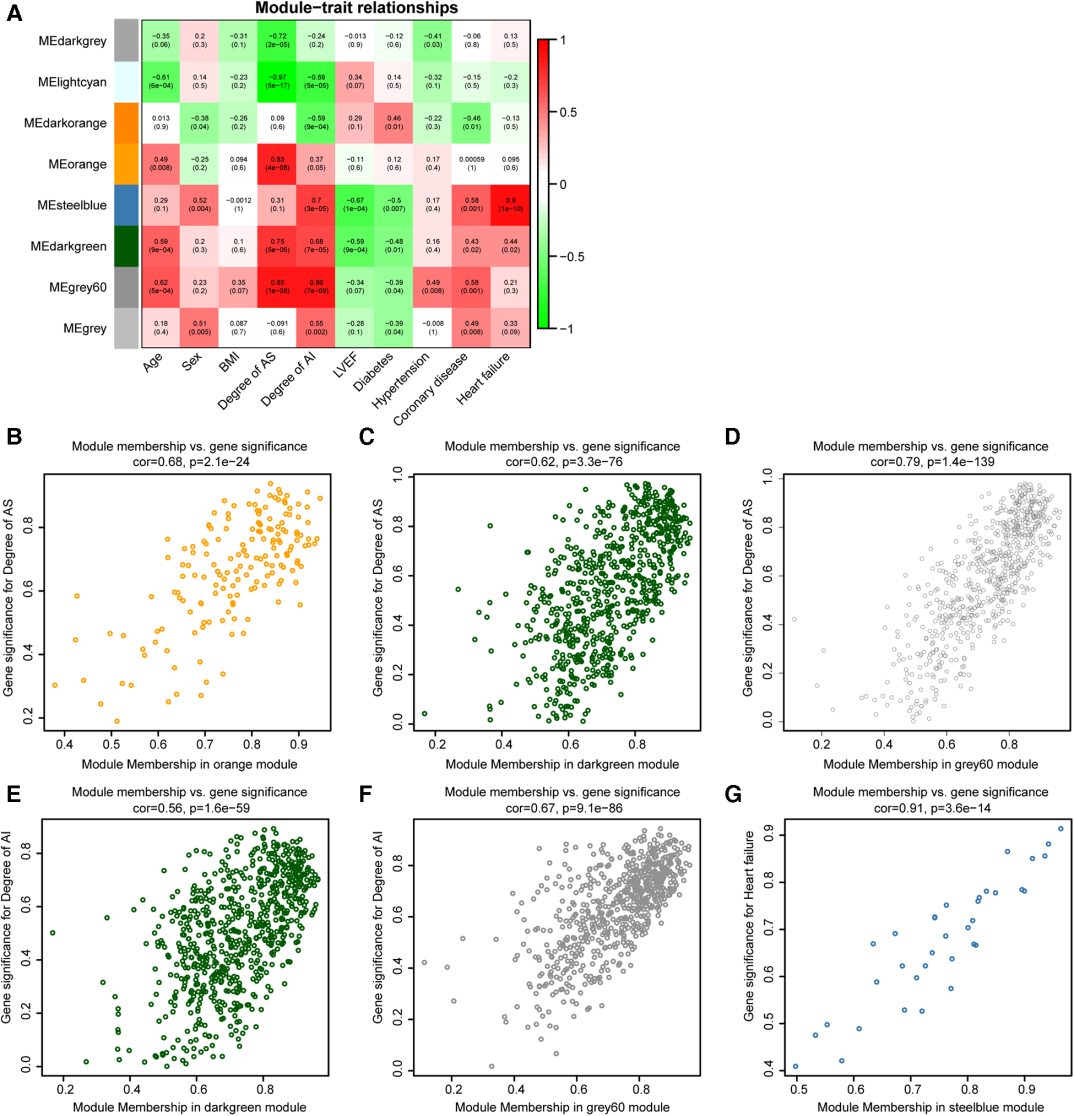
Identification of highly correlated modules connected with AS or AI. (**A**) The correlation heat map for various modules and clinical traits (age, sex, BMI, degree of AS, degree of AI, LVEF, diabetes, hypertension, coronary heart disease, and heart failure). Each cell contains the corresponding correlation coefficients and *P* value. (**B–G**) Scatterplot of Gene Significance (GS) for indicated clinical traits *vs.* Module Membership (MM) in the indicated modules.

PCA for gene expression values in the indicated modules were computed. The PC1 for indicated genes was defined as eigengene. The correlation analyses for modules and clinical traits were performed by eigengenes from various modules and clinical traits data of 28 samples (Figure 2A). To find the highly correlated genes with AS and AI pathophysiologic mechanisms, these modules (correlation coefficient >0.6 and *P* value <0.05) associated with the degree of AS, degree of AI, and heart failure were subjected to further analyses (Figure 2A).

To further study the relationship between modules and clinical traits, the associations of gene expression values with degree of AS, degree of AI, and heart failure were analysed, the correlation coefficients were defined as gene significances (GS). The correlations of gene expression values with the modules (orange, steelblue, darkgreen, and grey60 modules) screened out in Figure 2A were calculated, which were marked as module memberships (MM). The scatter plots for MM and GS in the indicated modules were plotted, these plots (module membership *vs.* gene significance) with correlation coefficients more than 0.5 and *P* value less 0.05 were regarded as highly correlated for MM and GS (Figures 2B–G). These plots were used to screen highly correlated genes in AS and AI pathogenesis. These genes with GS > 0.8, MM > 0.8, and *P* value <0.05 were singled out in the plots. There were 30, 108, 133, 29, 77, and 5 genes in indicated modules screened out by the pre-set parameters (Figures 2B–G). Finally, 302 highly correlated genes were identified after removing duplicates.

### GO and KEGG enrichment analysis for highly correlated genes

In order to parse the molecular functions involved by these 302 highly correlated genes, gene function enrichment analyses of GO and KEGG were conducted using clusterProfiler program package. The results of GO enrichment analyses including BP, MF, and CC were obtained. These terms of GO and KEGG enrichment analyses were ranked by ascending *P* value and descending gene counts. We selected top 10 highly correlated terms with AS and AI as significantly enriched terms in GO and KEGG analyses.

**Figure 3:**
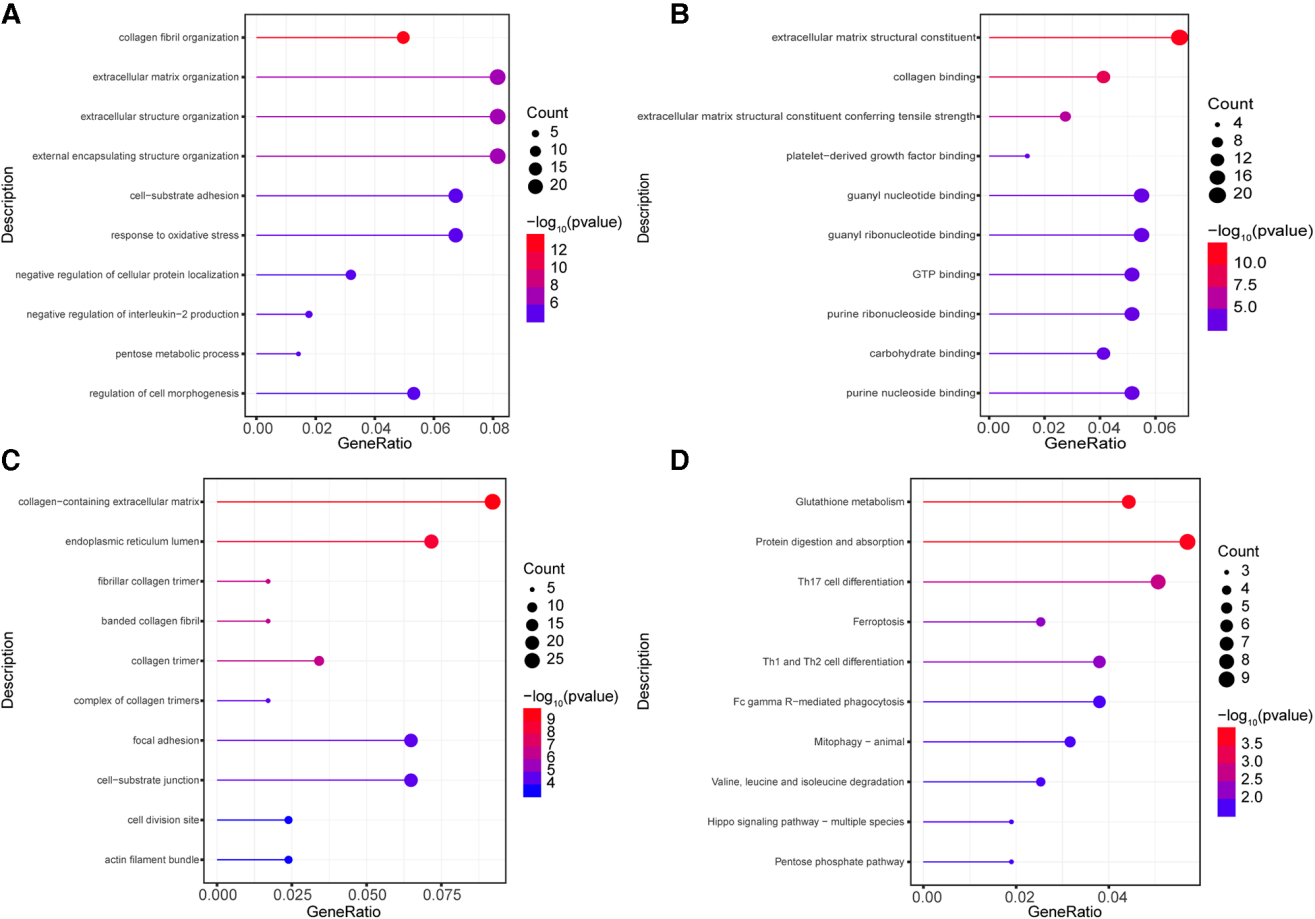
Go and KEGG enrichment analysis for highly correlated genes. (**A–C**) The GO enrichment analyses were conducted using the 302 highly correlated genes, the top 10 terms of from BP (**A**), MF (**B**), CC (**C**) were depicted. (**D**) The KEGG enrichment analyses were performed, the 10 significantly enriched pathways were identified. The circle size represents gene counts in each enriched term. The different color means significance for each enriched term.

Among these terms in BP analysis, our results showed that genes highly correlated with AS and AI were mainly enriched in collagen fibril organization (GO:0030199), extracellular matrix organization (GO:0030198), extracellular structure organization (GO:0043062), external encapsulating structure organization (GO:0045229), cell-substrate adhesion (GO:0031589), response to oxidative stress (GO:0006979), negative regulation of cellular protein localization (GO:1903828), negative regulation of interleukin-2 production (GO:0032703), pentose metabolic process (GO:0019321), and regulation of cell morphogenesis (GO:0022604) (Figure 3A). For MF analysis, the enrichment analysis results mainly included extracellular matrix structural constituent (GO:0005201), extracellular matrix structural constituent conferring tensile strength (GO:0030020), and kinds of molecular binding (GO:0005518, GO:0048407, GO:0019001, GO:0032561, GO:0005525, GO:0032550, GO:0030246, GO:0001883) (Figure 3B). In CC enrichment analysis, the significantly enriched terms mainly contained extracellular matrix (GO:0062023), endoplasmic reticulum lumen (GO:0005788), collagen (GO:0005583, GO:0098643, GO:0005581, and GO:0098644), focal adhesion (GO:0005925), cell-substrate junction (GO:0030055), cell division site (GO:0032153), and actin filament bundle (GO:0032432) (Figure 3C). These results indicated that the highly correlated genes mainly functioned in these courses.

To investigate the enriched pathways of these highly correlated genes, the KEGG enrichment analyses were conducted. As shown in Figure 3D, the significantly enriched pathways included protein digestion and absorption (hsa04974), glutathione metabolism (hsa00480), Th17 cell differentiation (hsa04659), Th1 and Th2 cell differentiation (hsa04658), ferroptosis (hsa04216), phagocytosis (hsa04658), mitophagy (hsa04137), valine, leucine and isoleucine degradation (hsa00280), hippo signaling pathway (hsa04392), and pentose phosphate pathway (hsa00030). These results revealed that these pathways may be important in the development of AS and AI.

### Identification of DEGs for highly correlated genes

**Figure 4:**
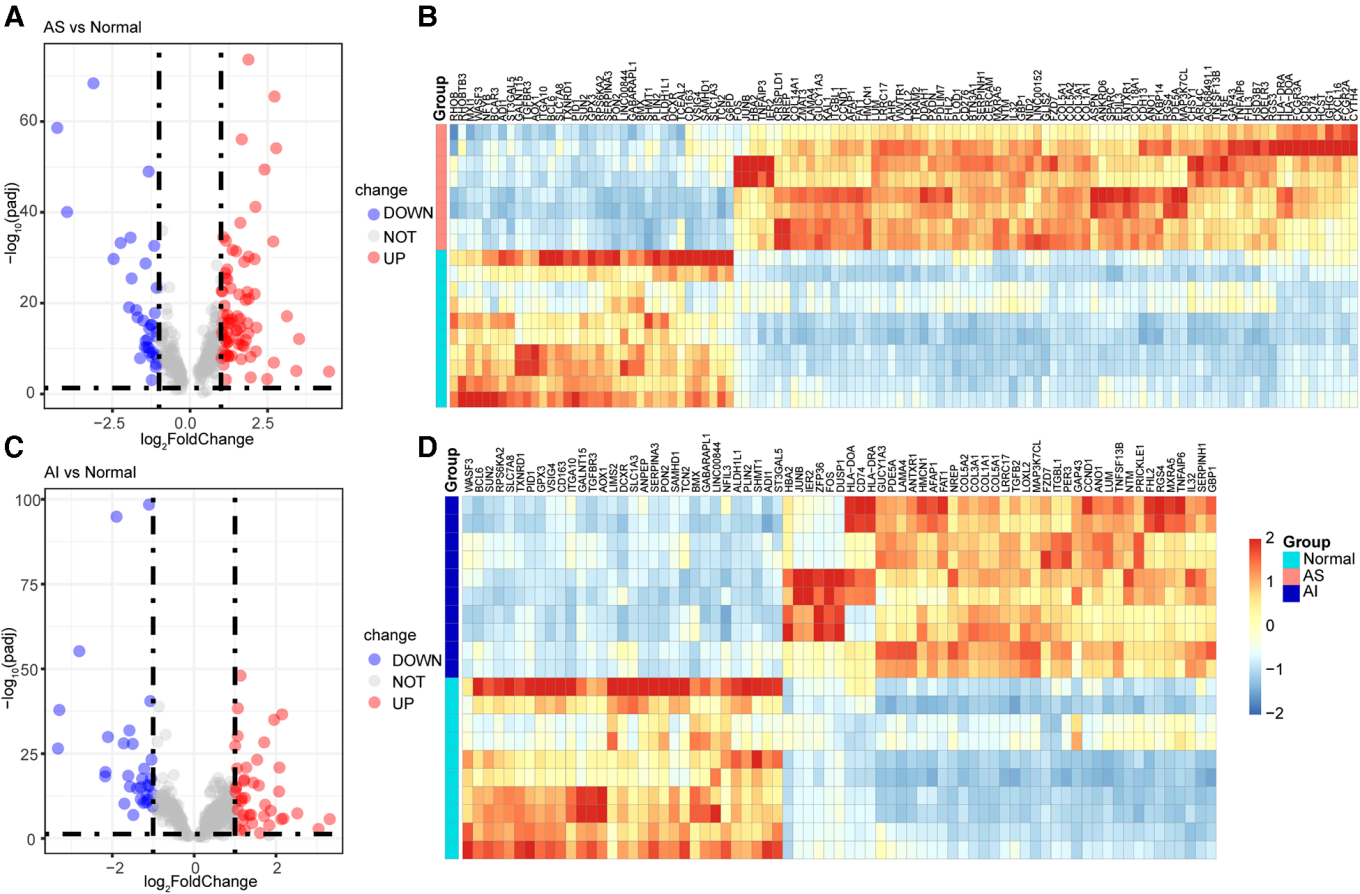
Identification of DEGs for highly correlated genes. (**A–D**) The 302 highly correlated genes with AS and AI were used for DEGs expression analyses. These genes with |log_2_FoldChange| ≥ 1 and adjust *P* value <0.05 were regarded as the DEGs in AS and AI group in comparison to the normal control group. The volcano plots and heatmaps were used for visualization of the DEGs for indicated group. The blue dots of volcano plots represent the downregulated genes (DOWN) in AS (**A**) and AI (**C**) group. The gray dots of volcano plots represent the unchanged genes (NOT) in AS (**A**) and AI (**C**) group. The red dots of volcano plots represent the upregulated genes (UP) in AS (**A**) and AI (**C**) group. The heatmaps of DEGs from (**A,C**) of AS (**B**) and AI (**D**) group were depicted, the color scale bar of heatmap represents the scale (from −2 to 2) for the expression levels of genes presented in the heatmaps with a breakpoint of zero represented by white.

To understand the expression of the 302 highly correlated genes in the AS and AI groups, we performed DEGs analyses using the DESeq2 package. These genes with |log_2_FoldChange| ≥ 1 and adjust *P* value <0.05 were considered as the DEGs. As depicted in Figures 4A–D, there were 31 downregulated genes, 229 unchanged genes, and 42 upregulated genes in the AI group and 35 downregulated genes, 190 unchanged genes, and 77 upregulated genes in the AS group in comparison to the control group. These differential expression profiles among AS, AI, and control group indicated the different molecular mechanism in AS and AI pathogenesis.

### Hub genes identification by PPI analysis

**Figure 5:**
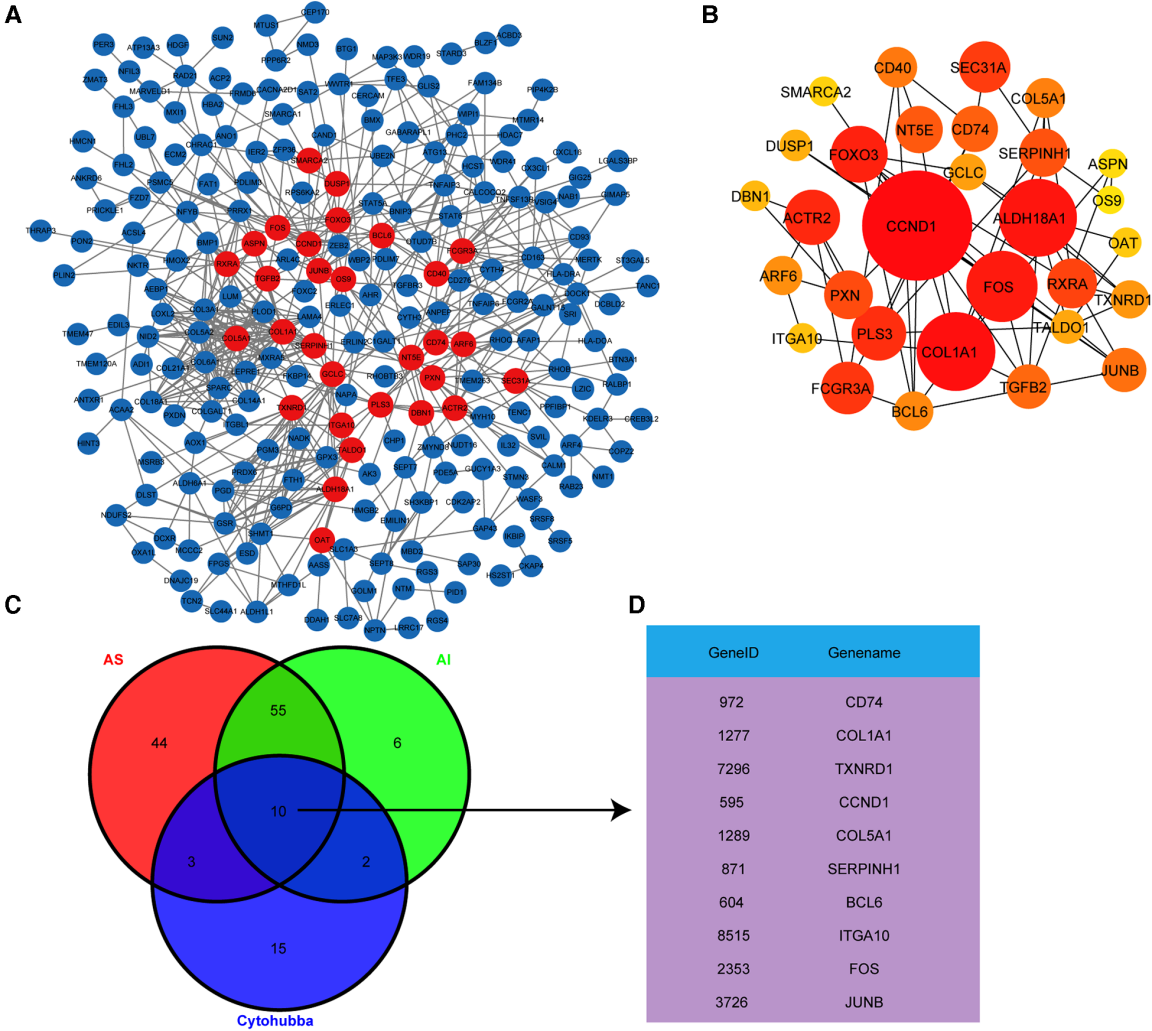
Hub genes identification by PPI analysis. (**A**) The PPI network analysis for the 302 highly correlated genes was conducted using the STRING online tool and visualized by Cytoscape software. Each node represents the gene, the different quantity of connecting line among these nodes represents connectivity. (**B**) The PPI network shown the top 30 genes with highest connectivity from (**A**) screened out with Cytohubba plug-in by Betweenness button, the node size represents different connectivity. (**C**) Venn diagram exhibited the overlapping of the DEGs from group and the top 30 genes with highest connectivity from (**B**) to screen out the hub genes. (**D**) These hub genes were exhibited in the gene list.

Based on the above results, we next explored the important hub genes with high connectivity among these highly correlated genes. The interaction network for the 302 highly correlated genes with AS and AI pathogenesis was constructed and visualized utilizing the PPI analysis tool STRING and Cytoscape, respectively (Figure 5A). To screen out these genes with highest connectivity, the recognized plug-in Cytohubba of Cytoscape software was used to select the top 30 genes with the highest connectivity among the 302 genes by Betweenness button (Figure 5B). To identify the functionally crucial hub genes in mediating AS and AI common pathological processes, we overlapped the DEGs from AS group, AI group, and the top 30 highly connected genes (Figures 5C,D). Finally, ten hub genes including *CD74*, *COL1A1*, *TXNRD1*, *CCND1*, *COL5A1*, *SERPINH1*, *BCL6*, *ITGA10*, *FOS*, and *JUNB* were obtained according to above-mentioned analytical methods (Figures 5C,D). These findings indicate that AS and AI shared the common molecular regulatory network.

### Validation of hub genes expression levels

**Figure 6:**
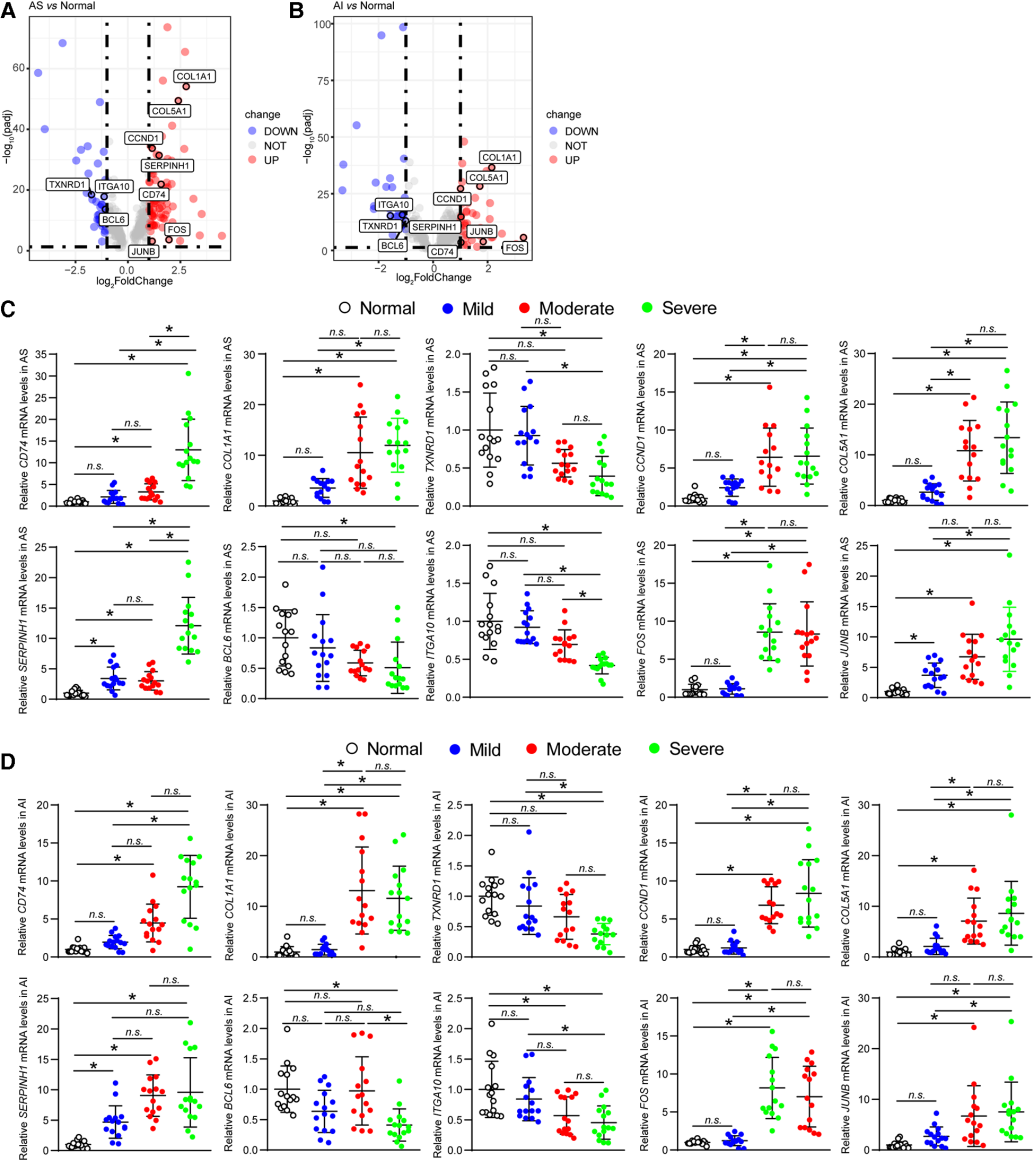
Validation of hub genes expression levels. (**A,B**) The mRNA expression levels of the ten hub genes (*CD74*, *COL1A1*, *TXNRD1*, *CCND1*, *COL5A1*, *SERPINH1*, *BCL6*, *ITGA10*, *FOS*, and *JUNB*) of AS and AI group were analysed using the expression values from the high throughput RNA-sequencing data. These genes were marked in the indicated volcano plot. (**C,D**) The mRNA expression levels of the ten hub genes (*CD74*, *COL1A1*, *TXNRD1*, *CCND1*, *COL5A1*, *SERPINH1*, *BCL6*, *ITGA10*, *FOS*, and *JUNB*) of AS and AI group were detected by real-time PCR assay using the human aortic valve samples of AS and AI. The mRNA levels were normalized to those of glyceraldehyde-3-phosphate dehydrogenase (*GAPDH*) (*n* = 5). *P < 0.05 *vs.* the normal control group, *n.s.*, no significance.

For further verifying the results acquired from above analyses, we first investigated the expression levels of the ten hub genes in AS and AI group by analyzing expression values in high throughput RNA-sequencing data. The expression levels of ten hub genes were re-analysed. The mRNA expression levels of *CD74*, *COL1A1*, *CCND1*, *COL5A1*, *SERPINH1*, *FOS*, and *JUNB* were obviously upregulated, whereas those of *TXNRD1*, *BCL6*, and *ITGA10* were evidently downregulated in AS and AI aortic valves relative to the normal controls (Figures 6A,B). Meanwhile, we selected 5 normal aortic valves, 15 AS aortic valves, and 15 AI aortic valves. These diseased aortic valves were divided into mild, moderate, and severe groups according to the degree of aortic valves lesion, respectively. The mRNA expression levels of *CD74*, *COL1A1*, *TXNRD1*, *CCND1*, *COL5A1*, *SERPINH1*, *BCL6*, *ITGA10*, *FOS*, and *JUNB* were detected. As shown in Figures 6C,D, although there was not evident degree of lesion-dependent trend, the mRNA expression levels of the ten hub genes were consistent with the results in Figures 6A,B from high-throughput sequencing. These highly consistent data suggest that the vital function of the ten hub genes in regulating and indicating the disease course for AS and AI.

## Discussion

Herein, using the published high throughput RNA-sequencing data (GSE153555) for AS and AI aortic valves, we conducted WGCNA to identify the key co-expression modules and hub genes involved in AS and AI pathogenesis. Total of eight modules including darkgrey, lightcyan, darkorange, orange, steelblue, darkgreen, grey60, and grey modules were constructed. The associations of modules and clinical traits were computed, and these modules associated with the development and outcome of AS and AI were further analysed to screened out the highly correlated genes with AS and AI pathogenesis. The 302 highly correlated genes were obtained from the indicated modules, and GO and KEGG functional enrichment analyses were performed to explore the potential biological processes and signaling pathways involved in these genes. Furthermore, the expression profiles of the 302 highly correlated genes were used for DEGs analyses. There were 31 downregulated genes, 229 unchanged genes, and 42 upregulated genes in AI group and 35 downregulated genes, 190 unchanged genes, and 77 upregulated genes in AS group, respectively. The PPI network analyses were performed and visualized by STRING online tool and Cytoscape software. The top 30 genes with highest connectivity were singled out and intersected with the DEGs in AS and AI group to find out the common functional hub molecules in AS and AI pathogenesis. Ultimately, ten hub genes (*CD74*, *COL1A1*, *TXNRD1*, *CCND1*, *COL5A1*, *SERPINH1*, *BCL6*, *ITGA10*, *FOS*, and *JUNB*) were obtained and the validation of expression levels was performed to elucidate the molecular mechanism of AS and AI pathological processes.

By exploiting WGCNA module construction, we screened seven significant gene modules associated with AS and AI. We selected highly correlated modules with AS and AI for further analysis, and the 302 highly correlated genes were picked out. Our study found that these highly correlated genes were mainly implicated in the regulation of collagen fibril and extracellular matrix (ECM) by GO analyses, which was consistent with the previous studies (22, 23). These results indicate that the disorders of collagen fibril and ECM may largely impact the normal function of aortic valves. According to the results of KEGG analyses, we enriched top 10 pathways for AS and AI affected mechanism, which included nutrient metabolism, T cell differentiation, ferroptosis, phagocytosis, mitophagy, and Hippo signaling pathway. Glutathione metabolism is the pivotal pathophysiological course for anti-oxidative stress and anti-aging (24). We identified that glutathione metabolism pathway was the significantly enriched term, this hinted that oxidative stress response could be the vital molecular mechanism in regulating the development of AS and AI. Our analysis results were similar with those of David R. A. Reyes et al. (25) and Michael Mahmoudi et al. (26), which manifested the highly reliability of our study. Besides, our study uncovered Th17 cell differentiation and Th1 and Th2 cell differentiation pathway were associated with AS and AI pathogenesis. As the distinctly important contributors for orchestrating adaptive immune responses, Th1, Th2, and Th17 cells are responsible to various intracellular or extracellular pathogens as well as organ-specific autoimmunity, which were activated by a series of cytokines (27, 28). Further, Immune Cell Abundance Identifier (ImmuCellAI) (29) is introduced for precisely estimating the abundance of immune cell types from the high throughput RNA-sequencing data (GSE153555). As shown in Supplementary Figure S2, the box plots demonstrated that AS and AI patients had a higher level of cytotoxic T cells, gamma delta T cells (γδ T), iTreg, Th2 and Tr1 and a lower level of macrophages, neutrophils, and Th17. Our findings indicated that tackling immune responses may become a possibility for harnessing the pathogenesis of AS and AI. Another, we identified the other significantly enriched pathways as the AS and AI underlying mechanisms, such as ferroptosis, phagocytosis, mitophagy, and Hippo signaling pathway. However, the detailed and direct functions in AS and AI for these identified pathways still needed for investigation deeply in the future.

To find out the important regulatory and indicative molecules for AS and AI, we focused on the 302 genes for further analysis. By DEGs analysis and screening these genes with high connectivity, we found out ten hub genes including *CD74*, *COL1A1*, *TXNRD1*, *CCND1*, *COL5A1*, *SERPINH1*, *BCL6*, *ITGA10*, *FOS*, and *JUNB*. In terms of the expression levels for the ten hub genes, our results manifested that *CD74*, *COL1A1*, *CCND1*, *COL5A1*, *SERPINH1*, *FOS*, and *JUNB* were significantly up-regulated. Whereas those of *TXNRD1*, *BCL6*, and *ITGA10* were obviously down-regulated. Although, transcriptional profiles of AS and AI pathological processes have been well parsed by Christina L. Greene et al. (14), the identification of highly correlated genes with the progression of AS and AI by systematic computerized algorithm remains unimplemented. Greene et al. used DEGs analysis only to screen for genes of interest, our analysis strategy may be more comprehensive and diverse. Our study may provide the relatively reliable molecular markers for mechanism researches, diagnosis, and treatment of AS and AI.

CD74 (MHC class II invariant chain, Ii), is a kind of type II transmembrane glycoprotein (30). CD74 functions in multiple biological processes and disease types, including lung adenocarcinoma (31), kidney disease (32), spondyloarthritis (33), colitis (34), etc. Collagen type I alpha 1 chain (COL1A1) and collagen type V alpha 1 chain (COL5A1) are the members of collagen family (35), mainly involved in various courses of tumor development, such as hepatocellular carcinogenesis and metastasis (36), immune infiltration in mesothelioma (37), metastasis of lung adenocarcinoma (38), tumor progression in ovarian cancer (39). Thioredoxin reductase 1 (TXNRD1) is a member of the thioredoxin system, regulating hepatocellular carcinoma (40), epilepsy (41), osteosarcoma (42). Cyclin D1 (CCND1) functions as a regulator of CDK kinases and regulates the cell-cycle during G1/S transition (43). Serpin family H member 1 (SERPINH1) is a member of the serpin superfamily of serine proteinase inhibitors and binds specifically to collagen, has been identified acting in gastric cancer metastasis (44) and proliferation and migration of retinal endothelial cells (45). B cell lymphoma 6 (BCL6) is a recognized sequence-specific transcriptional repressor and critical for regulating germinal centers homeostasis (46). Integrin subunit alpha 10 (ITGA10) is a receptor for collagen, has been supposed to be the prognostic biomarker for skin cutaneous melanoma and ovarian cancer (47, 48). Fos proto-oncogene, AP-1 transcription factor subunit (FOS) has been uncovered adjusting cell proliferation, differentiation, and transformation (49). JunB proto-oncogene, AP-1 transcription factor subunit (JUNB) is a member for AP-1 complex, has been identified as the cell proliferation inhibitor and senescence inducer (50), which involves in the regulation of oral squamous cell carcinoma (51) and osteoarthritis (52), etc. However, the molecular functions for these ten hub genes identified by our study in aortic valve were still unclear. Our study provided the feasible molecular bases for the mechanism research or clinical diagnosis and treatment targeting AS and AI, whereas the specific role for these molecules in AS and AI should be further confirmed using *in vitro* or *in vivo* experimental models of AS and AI. Collectively, we identified the key signaling pathways and hub genes (*CD74*, *COL1A1*, *TXNRD1*, *CCND1*, *COL5A1*, *SERPINH1*, *BCL6*, *ITGA10*, *FOS*, and *JUNB*) in AS and AI pathological processes, which may become the potential indicative biomarkers or important regulatory targets in AS and AI pathogenesis. Our findings probably provided the vital theoretical foundation for AS and AI study in the future.

## Data Availability

The datasets presented in this study can be found in online repositories. The names of the repository/repositories and accession number(s) can be found in the article/**Supplementary Material**.
