# Supplementary material for: Identification of hub genes and key signaling pathways by weighted gene co-expression network analysis for human aortic stenosis and insufficiency
Source: Front Cardiovasc Med. 2023 Aug 9;10:857578. doi: 10.3389/fcvm.2023.857578 (PMC10445149; doi:10.3389/fcvm.2023.857578)
Supplement: Supplementary file 1 [file Datasheet1.docx]

Supplementary Material

# Supplementary Figures

## Supplementary Figure S1
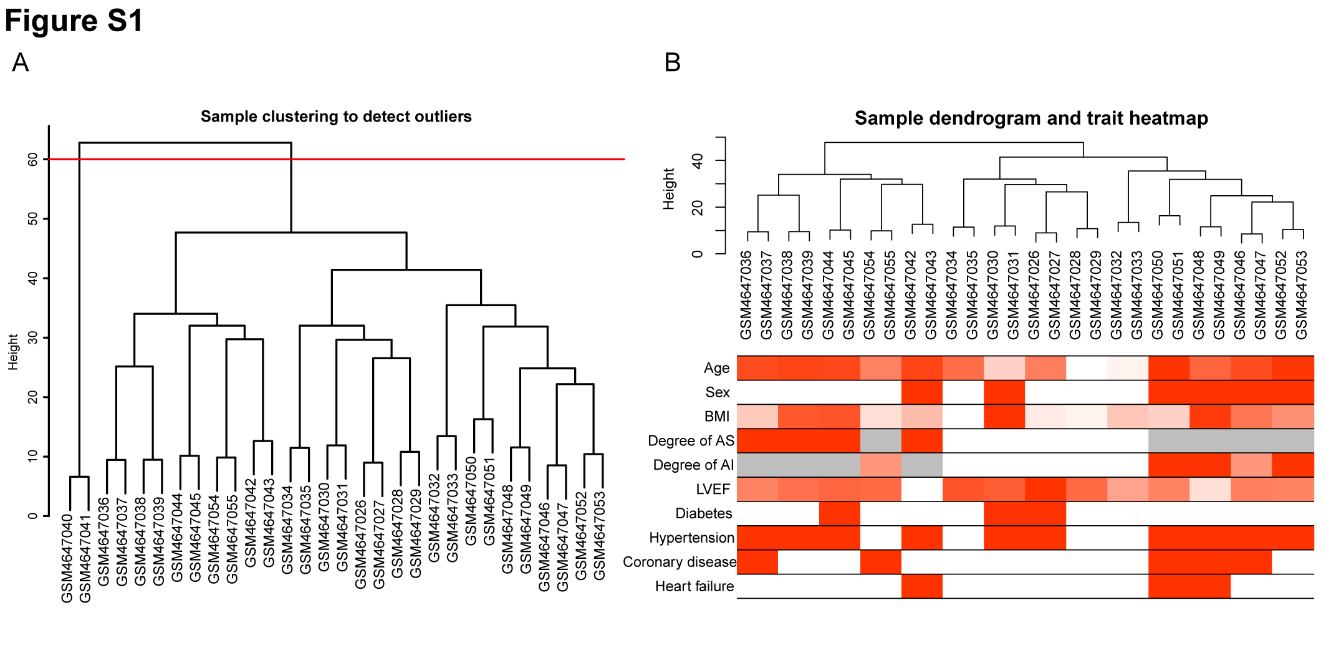


**Figure S1.** Data preprocessing before WGCNA co-expression modules construction. A, the hierarchical clustering analysis to find out and eliminate the outlier samples (GSM4647040 and GSM4647041). B, the sample dendrogram by clustering analysis and trait heatmap related to the sample dendrogram, white means a low value, red a high value, and grey a missing entry.

##
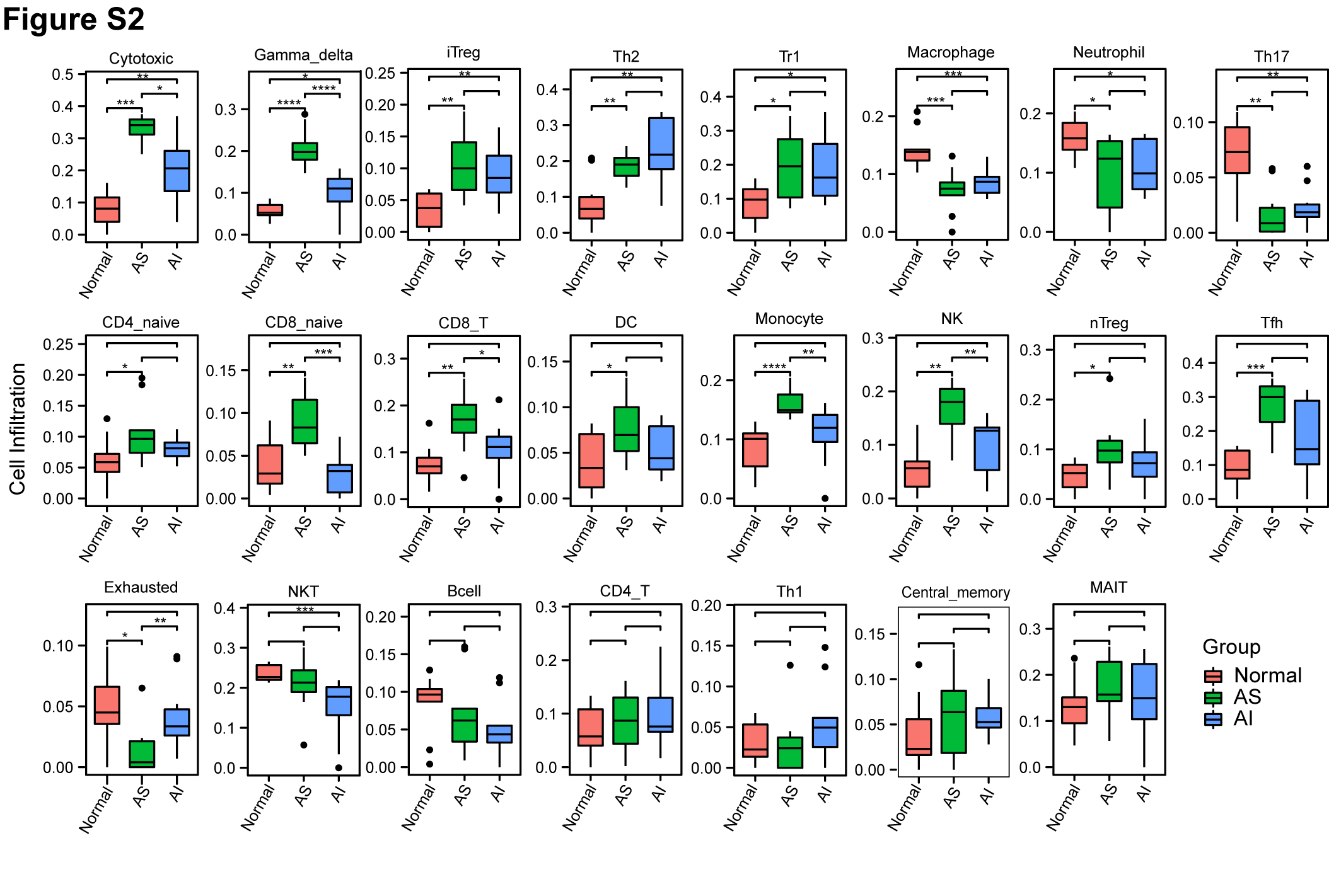
Supplementary Figure S2

**Figure S2,** Changes in the composition of infiltrating immune cells in aortic valve tissues. Box plots showing differences in the abundance of cytotoxic T cell (Tc), gamma delta T cell (γδ T), iTreg, Th2, Tr1, macrophage, neutrophil, Th17, CD4^+^ naïve T cell, CD8^+^ naïve T cell, CD8^+^ T cell, DC, monocyte, NK cell, nTreg, Tfh, exhausted T cell (Tex), natural killer T (NKT) cell, B cell, CD4^+^ T cell, Th1, central memory T cells (Tcm) and MAIT cell. **P* < 0.05, ***P* < 0.01, ****P* < 0.001 and *****P* < 0.0001 *vs* the normal control group.
